# Application of Magnetic Resonance Strain Analysis Using Feature Tracking in a Myocardial Infarction Model

**DOI:** 10.3390/tomography9020071

**Published:** 2023-04-18

**Authors:** Ryutaro Onishi, Junpei Ueda, Seiko Ide, Masahiro Koseki, Yasushi Sakata, Shigeyoshi Saito

**Affiliations:** 1 Department of Medical Physics and Engineering, Area of Medical Imaging Technology and Science, Division of Health Sciences, Osaka University Graduate School of Medicine, Osaka 565-0871, Japan; 2 Division of Clinical Radiology Service, Kyoto University Hospital, Kyoto 606-8507, Japan; 3 Department of Cardiovascular Medicine, Osaka University Graduate School of Medicine, Osaka 565-0871, Japan; 4 Department of Advanced Medical Technologies, National Cerebral and Cardiovascular Center Research Institute, Osaka 564-8565, Japan

**Keywords:** MR strain analysis, 7T-MRI, myocardial infarction model

## Abstract

This study validates the usefulness of myocardial strain analysis with cardiac cine magnetic resonance imaging (MRI) by evaluating the changes in the cardiac function and myocardial strain values longitudinally in a myocardial disease model. Six eight-week-old male Wistar rats were used as a model of myocardial infarction (MI). Cine images were taken in the short axis, two-chamber view longitudinal axis, and four-chamber view longitudinal axis directions in rats 3 and 9 days after MI and in control rats, with preclinical 7-T MRI. The control images and the images on days 3 and 9 were evaluated by measuring the ventricular ejection fraction (EF) and the strain values in the circumferential (CS), radial (RS), and longitudinal directions (LS). The CS decreased significantly 3 days after MI, but there was no difference between the images on days 3 and 9. The two-chamber view LS was −9.7 ± 2.1% at 3 days and −13.9 ± 1.4% at 9 days after MI. The four-chamber view LS was −9.9 ± 1.5% at 3 days and −11.9 ± 1.3% at 9 days after MI. Both the two- and four-chamber LS values were significantly decreased 3 days after MI. Myocardial strain analysis is, therefore, useful for assessing the pathophysiology of MI.

## 1. Introduction

Approximately 200,000 deaths are attributed to cardiac diseases reported annually in Japan, most of which comprise myocardial infarction (MI). MI is a disease in which plaque in the coronary arteries prevents the myocardium from obtaining sufficient blood flow, resulting in necrosis of the myocardium. Common symptoms include chest pain, chest tightness, and shortness of breath. However, approximately half of the patients with MI are asymptomatic and have no subjective symptoms. It is important to undergo an examination for early detection and treatment [1]. MI can be diagnosed using electrocardiography, hematology, echocardiography, or contrast-enhanced computed tomography (CT), but cardiovascular magnetic resonance (CMR) has recently attracted attention in the evaluation of MI and cardiac function.

CMR can calculate cardiac function by evaluating the left ventricular end-diastolic volume (LVEDV), the left ventricular end-diastolic volume (LVESV), and the left ventricular ejection fraction (LVEF), and it is widely used to assess cardiac function. Cardiac function assessment using CMR in rodents is as reproducible as that in humans [2] and has also been used to evaluate the pathogenesis of dilated cardiomyopathy and ischemic heart disease models [3,4,5]. CMR does not use radiation or contrast media. Therefore, this method can be repeatedly performed on the same animal. In preclinical studies, changes in the myocardium and cardiac function can be observed over time using CMR [6]. Furthermore, CMR is exceptionally accurate in assessing cardiac anatomy, perfusion, wall motion, and contractility, and its excellent soft-tissue contrast enables advanced myocardial tissue characterization and ventricular remodeling evaluation in animal models [7,8].

The parameters of local myocardial wall motion, such as longitudinal strain (LS), radial strain (RS), and circumferential strain (CS), can be quantitatively evaluated using echocardiography and magnetic resonance imaging (MRI). Speckle tracking echocardiography is the most common method for measuring myocardial wall motion because it is relatively inexpensive and widely applicable [9,10]. However, this method is greatly affected by the skill of the engineer and the setting of the machine, whereas magnetic resonance (MR) myocardial strain analysis, such as the tagging and harmonic phase (HARP) method, is highly reproducible [11,12]. These reports illustrate the accuracy of MR myocardial strain analysis in the diagnosis of coronary artery disease, myocardial ischemia, and systolic dysfunction [9,10,11,12]. Previous studies have shown that cardiac dysfunction reduces LS [13]. MR myocardial strain analysis using tagging and phase contrast has also been used to evaluate local myocardial wall motion in rodents [14,15]. However, these methods have a problem in that the examination time is extended since it is necessary to acquire a tagging image and a phase-contrast image. In recent years, attempts have been made to calculate myocardial strain from cardiac cine images [16,17].

In this study, cardiac cine imaging of MI model rats was acquired on days 3 and 9 after MI using 7-T MRI, and the cardiac function and myocardial strain values were calculated. The purpose of this study is to validate the usefulness of this method by evaluating the changes in cardiac function and myocardial strain values longitudinally in a myocardial disease model.

## 2. Materials and Methods

### 2.1. Animal Preparation

All the experimental protocols were approved by the Research Ethics Committee of Osaka University. All the experimental procedures involving animals and their care were carried out in accordance with the University Guidelines for Animal Experimentation and the National Institutes of Health Guide for the Care and Use of Laboratory Animals. The animals had free access to food and water and were kept in a room with a temperature of 23 °C and humidity below 50%. The animal experiments were performed using 8-week-old male Wistar rats (Japan SLC, Hamamatsu, Japan). There were six rats in the normal group (205.5 ± 6.9 g) and six in the group with left ventricular MI (184 ± 6.2 g). A total of 12 rats were used.

MI models were established by ligation of the left anterior descending coronary artery [18]. The model rats underwent tracheal intubation and were anesthetized with 3% isoflurane for induction and 1–2% isoflurane for maintenance of anesthesia. The left anterior descending coronary artery was ligated with a 7-0 polypropylene suture.

### 2.2. Magnetic Resonance Imaging

Cine MR images were taken in the rats at 3 and 9 days after MI and in the control rats. All the MRI was performed using a horizontal 7.0-T scanner (PharmaScan 70/16 US; Bruker, Ettlingen, Germany) equipped with a transmit/receive volume radiofrequency coil with a diameter of 60 mm. All the MRI experiments were performed under general anesthesia with 1.0–2.0% isoflurane (Abbott Laboratories, Abbott Park, IL, USA) administered through a mask covering the nose and mouth of the animals. The body temperatures were continuously maintained at 36.0 ± 0.5 °C by circulating water through heating pads throughout all the experiments. The respiratory signals and body temperature were monitored using a physiological monitoring system (SA Instruments, Stony Brook, NY, USA) [19,20]. Short-axis images were obtained using fast low-angle shots (FLASH) with navigator echo (IntraGate, Bruker) with the following parameters: repetition time (TR)/echo time (TE) = 6.4/1.4 ms, flip angle = 15°, movie frames = 15 frames per cardiac cycle, field of view (FOV) = 5.12 cm × 5.12 cm, acquisition time = 12 min 45 s, in-plane resolution per pixel = 270 µm, matrix = 192 × 192, number of excitations (NEX) = 300, oversampling = 250, and five concomitant slices covering the whole heart from the apex to the base. The long-axis four-chamber and long-axis two-chamber views were obtained using FLASH with navigator echo (IntraGate, Bruker) with the following parameters: TR/TE = 6.0/2.4 ms, flip angle = 10°, movie frames = 12 frames/s, FOV = 5.12 × 5.12 cm, acquisition time = 12 min 45 s, in-plane resolution per pixel = 313 µm, matrix = 192 × 192, NEX = 300, and oversampling = 250. The total scan time per animal was approximately 30 min.

### 2.3. MRI Data Analysis

The borders of the epicardium were manually outlined on the images of one cardiac phase of the short axis, four-chamber long axis, and two-chamber long axis. The LVESV, LVEDV, LVEF, right ventricular end-systolic volume (RVESV), right ventricular end-diastolic volume (RVEDV), and right ventricular ejection fraction (RVEF) were calculated from cine images of SA view stacks using cvi42 (Circle Cardiovascular Imaging, Calgary, Canada). In addition, strain analysis was performed by feature tracking using cvi42. For myocardial strain analysis, global RS and global CS using the short axis, and two LS values using the four-chamber long axis or two-chamber long axis, were calculated and statistically evaluated from images of the control rats and the rats at 3 and 9 days after MI.

### 2.4. Statistical Analysis

The LVESV, LVEDV, LVEF, RVESV, RVEDV, RVEF, and each strain value calculated from the MR images are presented as the mean ± standard deviation. All the statistical analyses were performed using Prism version 9 (GraphPad Software, San Diego, CA, USA). The differences were compared using one-way analysis of variance and Tukey’s multiple comparison test. The statistical significance was set at *p* < 0.05 (* p < 0.05, ** *p* < 0.01, *** *p* < 0.001, **** *p* < 0.0001).

## 3. Results

### 3.1. Observation with Cine Imaging

**Figure 1:**
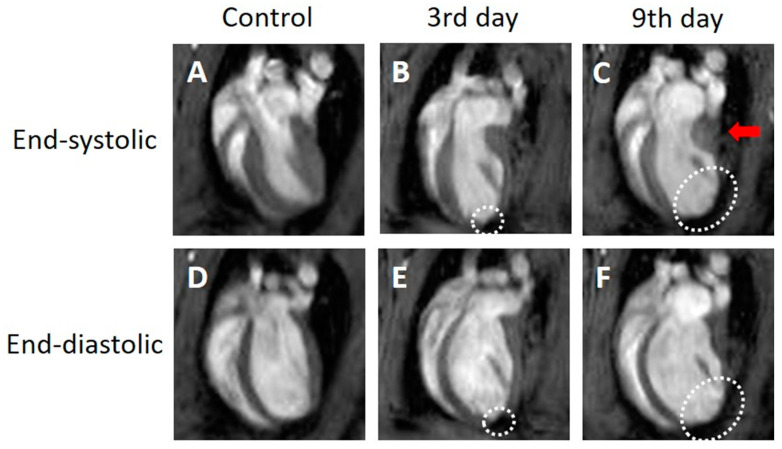
Representative four-chamber view cine magnetic resonance images. Control (**A**,**D**), 3 days after the onset of myocardial infarction (**B**,**E**), 9 days after the onset of myocardial infarction (**C**,**F**). End-systolic phase of the rat heart (**A**,**B**,**C**), end-diastolic phase of the rat heart (**D**,**E**,**F**). White dotted circles: infarcted area. Red arrow: thickened area.

In the four-chamber view cine images, the contraction and expansion of the entire myocardium were observed in the control group. Three days after MI, the end-systolic image (Figure 1B, white dotted circle) showed thinning of the infarcted myocardial wall at the apex of the side wall. Compared with the control group, there was an increase in the LVESV and no noticeable change in the LVEDV in the images taken 3 days after MI. The thinning of the myocardial wall further progressed from day 3 to day 9 after MI. The end-systolic (Figure 1C, white dotted circle) and end-diastolic images (Figure 1F, white dotted circle) showed thinning of the myocardial wall. As the myocardial thinning at the infarct region progressed, the end-systolic images 9 days after MI (Figure 1C, red arrow) showed thickening of the normal myocardial wall near the mitral valve.

**Figure 2:**
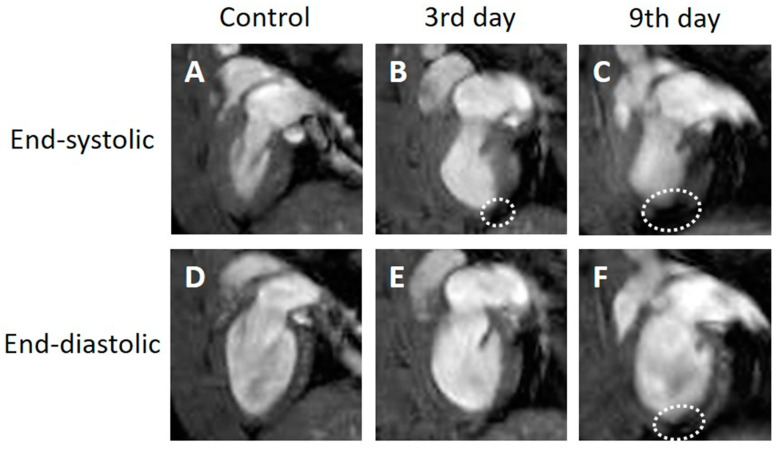
Representative two-chamber view cine magnetic resonance images. Control (**A**,**D**), 3 days after the onset of myocardial infarction (**B**,**E**), 9 days after the onset of myocardial infarction (**C**,**F**). End-systolic phase of the rat heart (**A**,**B**,**C**), end-diastolic phase of the rat heart (**D**,**E**,**F**). White dotted circles: infarcted area.

In the two-chamber view cine images, the contraction and expansion of the entire myocardium were observed in the control group. Three days after MI, the end-systolic images (Figure 2B, white dotted circle) showed thinning of the infarcted myocardial wall at the apex. An increase in the LVESV and a slight increase in the LVEDV were observed at 3 days after MI compared to the control group. No changes in the thinned myocardial wall were observed between days 3 and 9 after MI. However, increases in both the LVESV and LVEDV were observed 9 days after MI compared to the values 3 days after MI. Unlike the four-chamber view, the two-chamber view did not depict a clear thickening of the normal myocardial wall on day 9 (Figure 2C).

**Figure 3:**
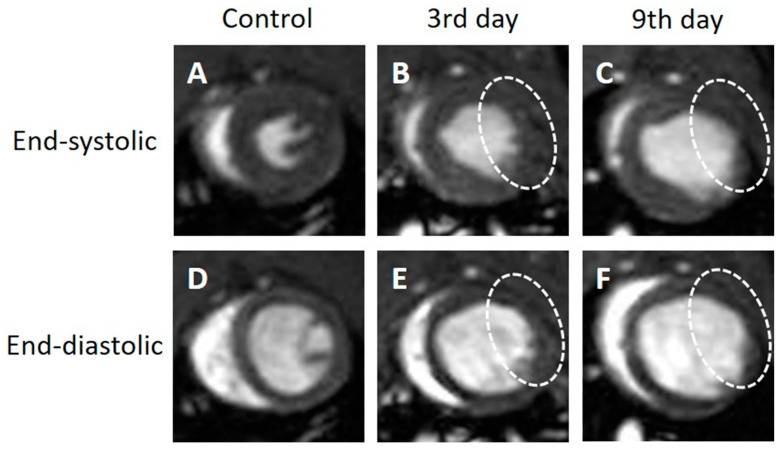
Representative short-axis view cine magnetic resonance images. Control (**A**,**D**), 3 days after the onset of myocardial infarction (**B**,**E**), 9 days after the onset of myocardial infarction (**C**,**F**). End-systolic phase of the rat heart (**A**,**B**,**C**), end-diastolic phase of the rat heart (**D**,**E**,**F**). White dotted circles: infarcted area.

In the short-axis cine images, the contraction and expansion of the entire myocardium were observed in the control group. At 3 days after MI, the end-systolic images (Figure 3B, white dotted circle) showed thinning of the infarcted myocardial wall on the side, with increases in both the LVESV and LVEDV compared to the control group (Figure 3B,E). The thinning of the myocardial wall further progressed from day 3 to day 9 after MI. The end-systolic (Figure 3C, white dotted circle) and end-diastolic images (Figure 3F, white dotted circle) showed thinning of the myocardial wall. The short-axis view did not show a clear thickening of the normal myocardial wall on day 9 (Figure 3C).

### 3.2. Comparison of Quantitative Values

**Figure 4:**
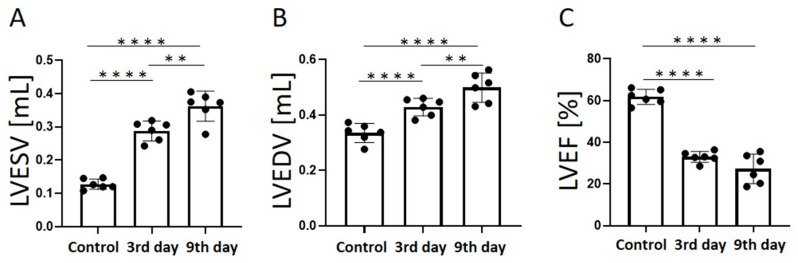
Graphs quantifying LVESV (**A**), LVEDV (**B**), and LVEF (**C**) in the control group, 3 days after the onset of myocardial infarction, and 9 days after the onset of myocardial infarction. LVESV, left ventricular end-systolic volume; LVEDV, left ventricular end-diastolic volume; LVEF, left ventricular ejection fraction. ** *p* < 0.01, **** *p* < 0.0001.

The LVESV (Figure 4A) increased significantly with time after the onset of MI (control, 0.1 ± 0.02 mL; day 3, 0.3 ± 0.03 mL, *p* < 0.0001; day 9, 0.4 ± 0.05 mL, *p* < 0.01). The LVEDV (Figure 4B) also increased significantly with time after the onset of MI (control, 0.3 ± 0.03 mL; day 3, 0.4 ± 0.03 mL, *p* < 0.0001; day 9, 0.5 ± 0.05 mL, *p* < 0.01). The LVEF decreased significantly 3 days after MI compared to the control group (control, 61.7 ± 3.6%; day 3, 33.0 ± 2.6%, *p* < 0.0001; day 9, 27.3 ± 7.1%, *p* < 0.0001), but there was no significant difference between days 3 and 9.

**Figure 5:**
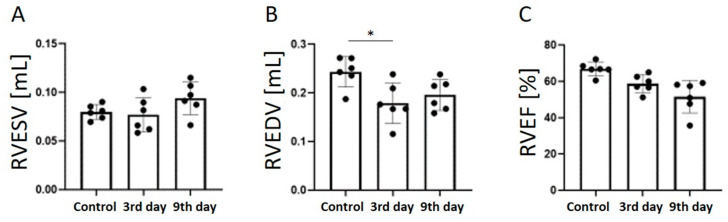
Graphs quantifying RVESV (**A**), RVEDV (**B**), and RVEF (**C**) in the control group, 3 days after the onset of myocardial infarction, and 9 days after the onset of myocardial infarction. RVESV, right ventricular end-systolic volume; RVEDV, right ventricular end-diastolic volume; RVEF, right ventricular ejection fraction. * p < 0.05.

The RVESV (Figure 5A) did not change significantly after the onset of MI, but it increased slightly on day 9 (control, 0.08 ± 0.01 mL; day 3, 0.08 ± 0.02 mL; day 9, 0.09 ± 0.02 mL). The RVEDV (Figure 5B) decreased significantly 3 days after the onset of MI compared to the control group (control, 0.2 ± 0.03 mL; day 3, 0.2 ± 0.04 mL, *p* < 0.05). There was no significant difference between days 3 and 9. The RVEF decreased significantly 9 days after MI compared to the control group (control, 66.9 ± 3.8%; day 3, 58.8 ± 4.9%; day 9, 51.5 ± 9.0%, *p* < 0.01).

### 3.3. Strain Analysis Results

**Figure 6:**
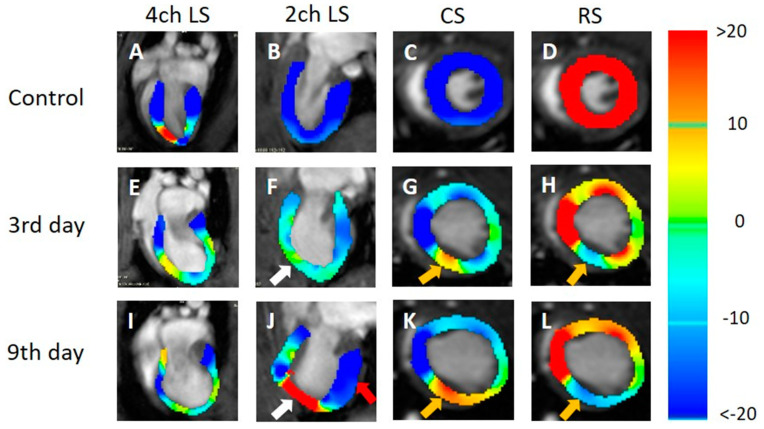
Strain-encoded functional magnetic resonance imaging of the end-systolic left ventricle. 4ch LS, four-chamber view longitudinal strain; 2ch LS, two-chamber view longitudinal strain; CS, short-axis view circumferential strain; RS, short-axis view radial strain. (**A**,**E**,**I**) LS in the long-axis four-chamber view. (**B**,**F**,**J**) LS in the long-axis two-chamber view. (**C**,**G**,**K**) CS in the short-axis view. (**D**,**H**,**L**) RS in the short-axis view. (**A**,**B**,**C**,**E**) Control, (**E**,**F**,**G**,**H**) 3 days after the onset of myocardial infarction, and (**I**,**J**,**K**,**L**) 9 days after the onset of myocardial infarction. The color bar shows the scale of the strain based on the end-diastolic left ventricle, with maximum contraction shown in red and minimum contraction in blue. White arrows: infarcted area. Red arrow: contracting area. Yellow arrows: reduced functionality.

In the strain analysis of the cine images of all the cross-sections (Figure 6A–L), the local strain value of the myocardium decreased on day 3 after MI compared with the control group. In the strain analysis of the 2ch-LS and short axis, the strain values of the apex and lower wall decreased at the left ventricular end-systole compared to those of the normal myocardium (Figure 6F–H,J–L). The tendency was strongly associated with the time from the onset and was remarkable on day 9 (Figure 6J–L). There was also a slight decrease in the strain value on the front wall. In the strain analysis of the 2ch-LS, the normal myocardium showed a high strain value on day 9 after MI compared to that on day 3.

**Figure 7:**
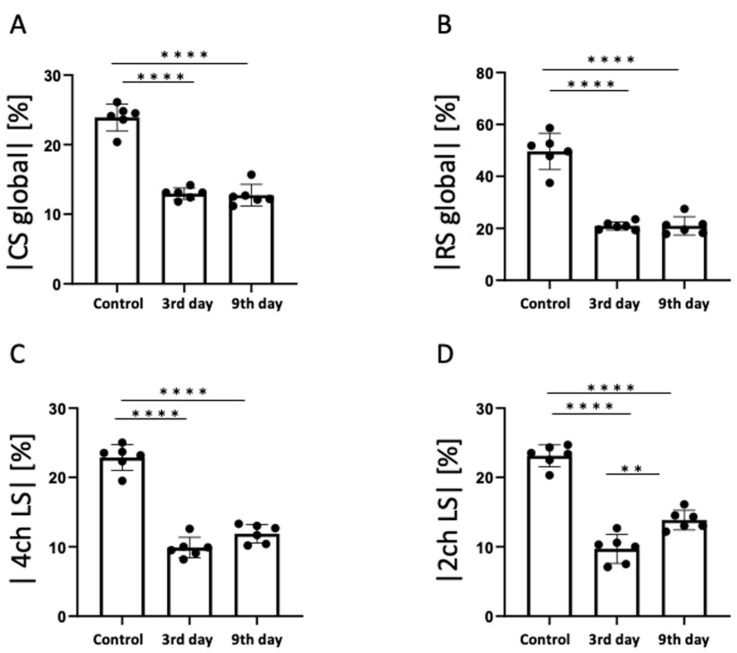
The results of statistical analysis of strain. (**A**) Global circumferential strain (CS), (**B**) Global radial strain (RS), (**C**) Four-chamber view longitudinal strain (4ch LS), (**D**) Two-chamber view longitudinal strain (2ch LS). ** *p* < 0.01, **** *p* < 0.0001.

According to the statistical analysis of the strain values, the global CS significantly decreased 3 days after MI compared to the control group (control, −23.9 ± 1.9%; day 3, −13.0 ± 0.8%; day 9, −12.7 ± 1.5%; Figure 7A). The RS value of the entire myocardium (global RS) also decreased significantly 3 days after MI compared with the control group (control, 49.7 ± 7.0%; day 3, 21.0 ± 1.6%; day 9, 21.0 ± 3.6%; Figure 7B). The 4ch-LS decreased significantly 3 days after MI compared to the control group. In The 4ch-LS, there was a slight upward trend from day 3 to day 9 (control, −22.9 ± 1.9%; day 3, −9.9 ± 1.5%; day 9, −11.9 ± 1.3%; Figure 7C). The 2ch-LS decreased significantly 3 days after MI compared to the control group (control, −23.1 ± 1.6%; 3 days, −9.7 ± 2.1%; *p* < 0.0001). However, the 2ch-LS 9 days after MI showed significantly higher values than those on day 3 (3 days, −9.7 ± 2.1%; 9 days, −13.9 ± 1.4%; *p* < 0.01; Figure 7D).

## 4. Discussion

In this study, cardiac cine imaging of MI model rats was acquired on days 3 and 9 after MI using 7-T MRI, and the cardiac function and myocardial strain values were evaluated. The cardiac function, such as the ejection fraction, in all three plane views decreased 3 days after MI. Both the 2ch- and 4ch-LS significantly decreased on day 3 after the onset of MI compared to the control group. However, the strain in the 2ch-LS view increased 9 days after MI. This suggests that a compensatory increase in myocardial motility in the non-infarcted myocardium might be detected as an increase in LS in our experiment. Strain analysis, which is superior to LVEF in assessing regional cardiac function and detecting early cardiac dysfunction, is rapidly gaining popularity in the field of CMR [21,22]. Our results reveal that myocardial strain analysis may be superior to ordinal parameters, such as end-systolic volume, end-diastolic volume, and ejection fraction for the assessment of regional cardiac function. Strain analysis by feature tracking, which allows for the evaluation of the strain values in three axes and volumes of both ventricles without additional sequences, is useful for the evaluation of acute remodeling in MI.

### 4.1. Cardiac Function of the MI Model

Cardiac function evaluation revealed a significant reduction in the LVEF. In a previous study, the LVEF of rats decreased by 30% 1–2 weeks after the onset of MI [23]. Our study results showed a 28.7% decrease in the LVEF on day 3 and a 34.4% decrease on day 9, which is consistent with the results of previous studies. Our results showed a significant increase in the LVESV with a decrease in the LVEF. Previous studies have shown that an increase in the end-systolic volume is correlated with a decrease in the ejection fraction [24], and the decrease in the LVEF in this study is considered to be mainly due to an increase in the LVESV. In previous studies, measurements were performed 1–2 weeks after the onset of MI [23], whereas this study revealed that LVEF decreases from day 3 after the onset of MI. LVESV is an important parameter in the evaluation of cardiac function using cardiac cine MRI. In a previous study using single-photon emission CT in a rat model of MI, it was shown that rats with a low ejection fraction and high end-systolic volume had more adverse cardiac events after MI [25]. Therefore, the measurement of left ventricular function, including LVEF and LVESV, is useful for diagnosing the degree of progression of MI. Cine MRI imaging using high-field 7-T MRI is useful for evaluating cardiac function in rodent MI models.

### 4.2. LS Analysis of the MI Model

The strain analysis in this study showed that the 2ch-LS of the whole myocardium increased significantly 9 days after MI. It has been reported that the myocardium of rats in a region distant from the infarction site becomes hyperactive 6 weeks after MI [26], suggesting that the increased motility of the non-infarcted myocardium compensates for hypokinesia of the infarcted myocardium. The authors evaluated the hyperfunction of non-infarct regions using the amount of work per unit length of the heart muscle. In contrast, a compensatory increase in myocardial motility in the non-infarcted myocardium was detected as an increase in the LS value in our experiment. Meanwhile, herein, we measured the strain values using feature tracking for the entire myocardium and obtained similar results to those of previous studies that measured the strain values for each region of the myocardium with receiver operating characteristic analyses. Our results showed a significant decrease in CS and RS values, which is consistent with the results reported by ROC analysis and the tagging method in previous studies [26,27].

Compared with existing methods, the method used in this study does not require additional imaging and allows the simultaneous calculation of cardiac function and strain values from cardiac cine images alone, making it a highly practical method in terms of the quantification and shortening of the examination time.

### 4.3. Limitations

In this study, changes in the myocardial wall motion during the subacute stage of MI were observed on days 3 and 9 after the onset of MI. Measurements were not performed before day 3 or after day 9. It is necessary to examine the changes in the myocardial strain and ventricular ejection fraction immediately after the onset and after day 9. We could have provided some information regarding the fibrotic process if we had observed it for a longer time. Observing changes in the myocardial wall motion during the hyperacute phase and after day 9 could be applied to the development and evaluation of treatment methods for MI.

Further observation of the timing at which LS begins to increase by measuring between days 3 and 9 can help to determine the period required for functional enhancement of the non-infarcted myocardium. Although six rats were used in this experiment, it is necessary to verify whether an increase in LS can be observed by the sample size. A previous study assessed the gene effect on myocardial deformation using cardiac magnetic resonance strain and elucidated its relationship to gene regulation and histology in a mouse heart failure model [28]. These results show that biochemical data, such as blood tests, myocardial tissue staining, and molecular biological evaluation, may help to evaluate the changes in pathological conditions in more detail. It is also necessary to evaluate the validity of the strain values and clarify the relationship with these measurement parameters by comparing them with the results of the strain analysis by echocardiography and the quantitative measurement of relaxation time by MRI [29]. In this study, strain analysis was performed for the entire myocardium, but segmental strain analysis should be performed for the subendocardial, central, and epicardial muscles. In the strain analysis based on cine MRI, standardization of the manual contouring of the heart muscle and automated evaluation of the tracking quality is needed in order to allow segments with lower image quality to be excluded from the analysis. Strain analysis based on cine MRI cannot be observed in real-time in comparison with echocardiography. Echocardiography has the advantage that when the operator would like to change the cross-section, they can do so in real-time. However, MRI is still not a robust method for real-time cardiac imaging. In addition, special software is required for MRI strain analysis. It cannot be performed on an operating console and requires a dedicated workstation. There are some improvements to be made for this to become a routine method.

Heart rates and classical indices, such as the ejection fraction, have long been known to be negatively correlated [30]. In addition, it is known that heart rate changes in children during growth have an important impact on both systolic and diastolic myocardial strain. Therefore, to evaluate regional myocardial function in children, the heart rate at rest should be considered an important factor [31]. Therefore, it is important to perform MRI imaging with a constant heart rate.

## 5. Conclusions

This study found that strain analysis by feature tracking, which can evaluate the strain values in three axes and volumes of both ventricles without additional sequences, is useful for evaluating acute remodeling in MI. Furthermore, changes in normal myocardial contraction, which supplements the function of the infarcted myocardium, appear in the long-axis direction.

## Data Availability

The data presented in this study are available upon request from the corresponding author.
